#  Long Term Effects of Mindfulness on Quality of life in Irritable Bowel Syndrome 

**Published:** 2015-04

**Authors:** Saeedeh Zomorrodi, Seyed Kazem Rasoulzadeh Tabatabaie, Parviz Azadfallah, Naser Ebrahimidaryani, Mohamad Arbabi

**Affiliations:** 1 Tarbiat Modares university, Tehran, Iran; 2 Department of Psychology, Teacher-Trbiat Modares University, Tehran, Iran; 3 Department of Psychology, Tarbiat Modares University, Tehran, Iran; 4 University of Medical Sciences, Tehran, Iran; 5 Department of Psychiatry, Psychiatry and Psychology Research Centre, Tehran University of Medical Sciences, Tehran, Iran

**Keywords:** *Mindfulness-Based Therapy*, *Irritable Bowel Syndrome*, *Life Quality*.

## Abstract

**Objectives:** This study aimed to investigate the long-term effects of mindfulness-based therapy on improving life quality of patients with irritable bowel syndrome.

**Method**: This was an experimental study including 24 patients (12 from each group) with IBS syndrome were selected based on the ROMEIII criteria and were randomly placed in the test and control groups. In both groups, the scales of the IBS-QOL34 Questionnaire were applied as assessment tool. Experiment group was subjected to the MFT (mindfulness-based therapy), while the control group received no intervention. After the two-month follow up, both groups were once again evaluated through the IBS-QOL34 scales.

**Results**: There is not significant difference between trial and control group in starting of the study in demographic and quality of life status. The findings of covariance analysis revealed that the difference between the experiment and the control groups at follow-up was significant (p = 0.01). The results showed that the MFT has long-term effects on the life quality of patients suffering from IBS.

**Conclusion:** The MFT could be considered as a new, effective and stable method in psychotherapy, particularly in psychosomatic disorders such as Irritable Bowel Syndrome.

Of the vast spectrum of psychosomatic disorders that have so far been diagnosed, certain maladies such as cardio-vascular diseases have received more attention psychologically, whereas others such as gastrointestinal disorders have not. Due to the prevalence of gastrointestinal diseases/disorders, and the role of psychological factors in their occurrence, more research in this field seems necessary. Functional gastrointestinal disorders are a group of psychosomatic diseases characterized by unknown etiology, questionable diagnostic criteria, prolonged unpredictable periods of illness and little drug effects (1). Of the 25 known functional gastrointestinal disorders, IBS is the most prevalent, the most expensive to treat and the most debilitating (2). IBS is characterized by changes in bowel habits, pain and abdominal discomfort without any detected structural abnormalities. As there are no clear diagnostic markers for IBS, its diagnosis is based on clinical manifestations (3) The IBS symptoms tend to periodically disappear and recur and are often mistaken for other functional disorders including fibromyalgia, headache, backache, and urinary-reproductive symptoms. The intensity of these symptoms vary and can considerably disrupt the patient’s quality of life as well as cause heavy expenses for treatment. No etiology is yet found for IBS although the following have been cited in the literature: abnormal gastrointestinal motor and sensory activities, CNS dysfunction, psychological disorders and stress (3). Abnormal psychological symptoms have been recorded in about 80% of IBS patients, particularly in referral centers; however, no single dominant psychiatric diagnosis was found (3). The psychological causes of gastrointestinal diseases encourage us to primarily discuss the psychological roots of IBS, which is the most common of these diseases. In addition, a significant relationship was found between IBS symptoms/anxiety level and the experience of sexual and physical abuse in childhood (2, 3). In functional gastrointestinal disorders, as there are no reliable objective/clinical symptoms and as these symptoms are not significant or justifiable to patients, the “health-related quality of life” is measured to determine the extent of therapeutic effects as well as the level of disruption caused in the patients’ physical, psychological and social routines (6). Research shows that health-related life quality of IBS patients is considerably low and is only comparable to that of diabetics and heart failure/defect patients who have a high mortality rate. For IBS patients, various aspects of life such as professional performance, travel, relationships with others and sense of pleasure are disrupted (7). High levels of stress, anxiety and worry reduce life quality for these patients (8). These patients have an adverse quality of life compared to healthy people. Compared to the normal group, IBS patients have 3 times more cases of absence from work (5) and they have higher medical expenses according to reports (3). For this reason, health-related quality of life can be used as a measure to determine the degree of reduced symptoms as well as improved health and social-psychological performance; thus making the improvement of life quality into the most important goal in the treatment of IBS patients (6). The understanding of IBS pathology and therapy has, within the past 30 years, been transformed from a simplistic reductionist view into a complex biological-psychological-social syndrome where behavioral and cognitive aspects as well as their interactions are considered in addition to genetic and physiological knowledge about the digestive system.

Considering the effect of IBS on psychological factors, we can conclude that psychological treatments should be applied to improve the life quality as well as reduce the symptoms of IBS patients. A wide spectrum of psychological treatments have been used for IBS, including hypnotherapy (9), bio feedback (9,10), cognitive behavioral therapy (CBT) (individually and in groups) (2,9,11,12,13), relaxation training (9,14,15) and dynamic interpersonal psychotherapy (16). For many years, the CBT was considered as the most effective form of psychotherapy for IBS (17). In the 1980s and 1990s in particular, CBT was applied as an effective therapy for reduction of IBS symptoms (5). In the IBS cognition model, it is assumed that dysfunctional thoughts and the cognitive process (thinking, memory, and attention) act through establishing neural connection between the brain and the bowel in biological systems, disrupting emotions as well as bowel functions and leading to IBS symptoms (18). In spite of the positive role of CBT in IBS treatment, the CBT literature does not report any long-lasting effects, and that is why IBS treatment through CBT has only demonstrated short-term effects (5, 19). 

According to the results obtained from follow-up studies, not only have cognitive – behavioral therapies been ineffective on IBS patients (5,19), but also two comprehensive studies suggest that CBT on the whole has failed to produce any positive results in IBS treatment (5). Considering the contradictory results recently obtained in CBT treatment, and the nonexistence of a lasting effect in the follow-up studies, it seems that CBT has been used in IBS cases mostly because it has been the dominant approach in recent decades, rather than having produced positive results in the particular case of IBS treatment. This points out to the fact that it might be helpful to consider other psychological interventions in the search for an effective approach for IBS treatment. The mindfulness-based cognitive method is the most recent method proposed for IBS therapy. However, little research has been conducted on the effectiveness of this method. 

The mindfulness-based therapy (MFT) claims to have long lasting effects on treatment of IBS patients (5, 20). Theoretically, mindfulness is related to psychological well-being and is defined as the instantaneous non-judgmental awareness and acceptance demonstrated by a person. Mindfulness can act as an antidote against common forms of psychological distress, i.e., avoidance, suppression or mental and emotional hyperactivity. Mindfulness-based interventions considerably reduce negative automatic thoughts and increase the ability of the patient to overcome such thoughts, bringing about psychological well-being (21). With respect to these and other similar results, and considering the significance of mindfulness-based psychotherapy in IBS treatment, particularly in prolonged and follow-up treatments, the authors employed this therapy to study the life quality improvement in IBS patients.

## Material and Methods

The present methodology was an experimental study with pretest, final test and two- month follow-up. 

*Statistical Population*

IBS patients referring to Imam Khomeini Hospital and a subspecialty private gastrointestinal clinic in Tehran, who diagnosed for IBS based on ROMEIII criteria participated in this study.

Sample Group and Sampling Method: 

With consideration of the experimental nature of the method used as well as the reduction of the participants, 24 patients (12 in each group) were selected as available samples and were randomly placed in the test and control groups.

*Inclusion criteria:*

-Age between 18 and 40

-An Associate degree as the minimum educational requirement (because of the active participation required in the therapy and nightly assignments, a certain level of motivation as well as ability in identifying their own intellectual and emotional states was needed)

-Inform consent and active participation in the study 

Therapeutic Mode

•Mindfulness-Based Therapy (MFT) for IBS Patients:

This therapeutic method was designed according to mindfulness method based on pain and stress management in IBS patients based on MBSR (mindfulness based on stress reduction) (13). The therapy was scheduled as eight 2-hour weekly sessions. 

-            Demographic Questionnaire (Researcher Made) included demographic information, i.e., age, sex, profession and educational background.

-            IBS-QOL-34 (Quality of Life) Questionnaire: This questionnaire was compiled by Patrick and Drassman in 1988. The coefficient of internal consistency in this questionnaire was equal to 0.96 (α = 0.96) and the correlation coefficient was significant with respect to intensity of disease symptoms (r = 0.67 and p<0.01). This questionnaire was validated in Iran by Ebrahimidaryani (2003). In the validation process, the internal consistency coefficients were obtained as α = 0.88 and n = 41. The validity of this questionnaire was investigated in the present study as well. The Alpha-Cronbach Method was used to calculate the value of α = 0.92. 

*Procedure*

Upon the selection of the sample group according to inclusion criteria, and upon the conduction of clinical and diagnostic examination according to ROMEIII criteria by a gastrointestinal specialist, 24 patients diagnosed with IBS were selected. The demographic researcher made the questionnaire, and the IBS patients’ quality of life questionnaire was filled in by the patients in the pretest stage. Ultimately, the therapy combined with drug treatment method was conducted on the test group, while the control group was only treated with drugs without psychotherapy. The test and control groups were subsequently evaluated via the IBS-QOL34 questionnaire in the post-test and the follow-up stages. 

*Data Analysis Methods*

In the present study, statistical inference methods including the One-Way Covariance Analysis were used in addition to descriptive methods to test the hypotheses made during testing. SPSS Software version 20 was used to analyze the obtained data.

## Results

The findings revealed that the total mean age of the patients was 34 years. There were 13 men and 11 women in the study; of whom, 15 were married and 9 were single. Most patients had associate or B.S. degrees (Table 1).

**Table 1:** Demographic Characteristics of Test Subjects according to Therapy Groups

**Variables**	**Mindfulness** **(12 Patients)**	**Control** **(12 Patients)**
**Groups**		
Age (Mean/Standard Deviation)	34/25±4/16	33/42±5/3
Sex: Women (No./Percent) Men (No./Percent)	6(50) 6(50)	5(48) 7(59)
Marital Status: Married (No./Percent) Single (No./Percent)	5(41) 7(51)	10(83) 2(17)
Academic Degree: Associate (No./Percent) B.S. (No./Percent) M.S. (No./Percent) Ph. D. (No./Percent)	6(50) 4(33) 2(17) 0(0)	4(33) 5(42) 2(17) 1(8)

**Table 2:** Descriptive results of the quality of life variable separated in terms of groups and time

		**Pre-test**		**Post-test**		**Follow up**	
Group	N	Mean	Mean Difference	Mean	Mean Difference	Mean	Mean Difference
Mindfulness	12	96.00	23.6	70.7	15.15	67.17	16.5
Control	12	113.08	13.2	108.3	12.9	114.9	11.6

**Table 3.1:** Results of One-Way Covariance Method

**Source of variance**	**Type of III some ** **square**	**df**	**Mean square**	**F**	**Sig**	**Observed ** **Power**
Total Q.O.L. pre	2295.4	1	2295.4	23.2	0.0001	0.835
Group	3991.1	1	3991.1	40.4	0.0001	1.000
Error	2075.5	21	98.8			
Total	204913	24				

**Table 3.2:** Pairwise Comparisons

**Group1**	**Group2**	**Mean Difference ** **(1-2)**	**Std. Error**	**Sig.** b	**95% Confidence Interval for Difference** b	
					Lower Bound	Upper Bound
Control	Mindfulness	28.459*	4.479	0.0001	19.146	37.773

Dependent Variable: total Q.O.L post

Based on estimated marginal means

* . The mean difference is significant at the .05 level.

b . Adjustment for multiple comparisons: Least Significant Difference (equivalent to no adjustments).

**Table 4.1:** The Results One-way Covariance Analysis for Post-Test Control

**Source of variance**	**Type of III some ** **square**	**df**	**Mean square**	**F**	**Sig**	**Observed ** **Power**
T otal Q.O.L. post	2955	1	2955	41.2	0.0001	1
Group	579.5	1	579.5	8.07	0. 01	0.773
Error	1507	21	71.8			
Total	217069	24				

**Table 4.2:** Pairwise Comparisons

**Group1**	**Group2**	**Mean Difference ** **(1-2)**	**Std. Error**	**Sig.** b	**95% Confidence Interval for Difference** b	
					Lower Bound	Upper Bound
Control	Mindfulness	16.848*	5.930	0.010	4.516	29.180

Dependent Variable: totalQ.O.L.falo

Based on estimated marginal means

* . The mean difference is significant at the .05 level.

b . Adjustment for multiple comparisons: Least Significant Difference (equivalent to no adjustments).

The descriptive results of the quality of life variable are separately demonstrated in Table 2 in terms of groups and time.

To test the hypotheses of the study, i.e., “Mindfulness-based therapy has prolonged effects on the life quality of IBS patients”, and considering that a pre-test was used, one-way covariance analysis was utilized. Therefore, first the assumptions of the analysis were checked out and approved. Then, the one-way covariance method was utilized. The results are presented in Tables 3.1 and 3.2.

The results of covariance analysis revealed that although the pre-test effect/control was significant, the difference between test and control groups was significant at p<0.001 (i.e., the effectiveness of MFT treatment at the post-test stage was confirmed). Post incidental comparisons also revealed a significant difference between the test and control groups.

In addition, another one-way covariance analysis was performed to control the post-test effect. The results of this analysis are presented in Tables 4.1 and 4.2.

As can be observed in the table, the results of covariance analysis revealed that due to the effect of the post-test at follow-up and controlling the same, the difference between the test and the control groups at follow-up was significant (p = 0.01). This means that the MFT therapy affects the patients’ quality of life in the long-run as well. The results obtained from the incidental test also showed a significant difference between the test and control groups 

Regarding the exerted control. Therefore, the main hypothesis of his study was confirmed. 

## Discussion

Health-related quality of life as a multi-dimensional concept can be used as a measure to reduce the symptoms and at the same time improve health and psychological-social function (6). The results of this research revealed that mindfulness-based therapy has sustainable prolonged effects on the life quality of IBS patients. These results are in agreement with those of other studies in this regard. Although very little research has so far been conducted in this area, it seems that the gap that used to exist previously due to lack of prolonged effect in other psychological treatments of IBS patients has finally been bridged. In fact, one of the most recent approaches in IBS treatment is the mindfulness-based CBT. Implementation of mindfulness-based approaches and ACT-based therapy can help reduce the physical, emotional and ultimately intellectual symptoms as well as experimental avoidance (18). Mindfulness techniques lead to improvement of life quality as well as effective coping methods, alleviated depression and reduced anger (20). Mindfulness intervention is accompanied by high levels of life satisfaction (24), success (25), self-confidence, optimism, quality of life and pleasant feelings (26). Evidence shows that mindfulness is related to mental health indexes such as high levels of positive affect, liveliness, adaptive emotional regulation, lower levels of negative affect and psychological symptoms (26). On the other hand, mindfulness-based techniques lead to reduced stress and pain symptoms (27). Mindfulness-based interventions have lead to reduced frequency of negative automatic thoughts and ultimately to psychological welfare (21). Evidence shows that mindfulness is related to psychological health indexes such as high levels of positive affect, liveliness and adaptive emotion regulation, as well as lower levels of negative affect and psychological symptoms (26). Mindfulness-based training and interventions lead to meta-cognitive awareness as well as reduction of mental rumination and negative thoughts (26). In the mindfulness-based meta-cognitional model in IBS therapy, it is assumed that experiences related to body feelings often cause anxiety. Anxiety-related bodily sensations and the subsequent attempt to avoid those sensations create gastrointestinal anxiety, e.g., social avoidance or avoiding professional success and finally lead to social isolation and depression (5).

Interaction between specific anxiety and avoidance behavior gives rise to the concept that the experienced avoidance can be the cause of constant suffering. Therefore, the first step in the meta-cognition model based on mindfulness is to increase meta-cognitional awareness (i.e., the ability to obtain a new cognition regarding personal intellects and affects, as well as looking upon these matters as transient events that would pass, rather than considering them to be established facts). Subsequently, this advanced awareness is assumed to reduce rumination. As a result, catastrophizing, mental rumination and other symptoms including stress as well as passive ineffective coping skills would also decrease (2,5,20), resulting in patients’ improved quality of life. Such meta-cognitional awareness can introduce more stable effects in patients and persist for long periods of time.

**Limitation**

There are some issues in this study like low sample size and no intervention in control group which limit generalization results of this experiment. New studies with larger sample and planning sham intervention could be more valid to apply in clinical practice.
